# Vertebral artery injury in major trauma patients in Saudi Arabia: A retrospective cohort study

**DOI:** 10.1038/s41598-020-73238-2

**Published:** 2020-10-01

**Authors:** Sharfuddin Chowdhury, Sadiq Hussain Almubarak, Khadega Hadi Binsaad, Biswadev Mitra, Mark Fitzgerald

**Affiliations:** 1 King Saud Medical City, Riyadh, Kingdom of Saudi Arabia; 2 Department of General Surgery, King Fahad Hospital, Al-Hofuf, Kingdom of Saudi Arabia; 3 Alfaisal University, Riyadh, Kingdom of Saudi Arabia; 4 National Trauma Research Institute, 89 Commercial Road, Melbourne, VIC 3004 Australia; 5 Alfred Health, The Alfred Hospital, 55 Commercial Road, Melbourne, VIC 3004 Australia; 6 Critical Care Division, Department of Epidemiology and Preventive Medicine, School of Public Health and Preventive Medicine, Monash University, Melbourne, Australia

**Keywords:** Diseases, Medical research

## Abstract

Blunt vertebral artery injury (VAI) is associated with severe cervicocephalic trauma and may have devastating consequences. This study aimed to determine the incidence and nature of VAI in polytrauma patients. The secondary objective was to assess the association of VAI with previously suggested risk factors. It was a retrospective observational study of all polytrauma patients admitted to the trauma unit between April 2018 and July 2019, who had CT neck angiography to diagnose blunt VAI according to modified Denver criteria. Out of 1084 admitted polytrauma patients, 1025 (94.6%) sustained blunt trauma. Of these, 120 (11.7%) underwent screening CT neck angiography. VAI was detected in 10 (8.3%; 95% CI 4.1–14.8) patients. There were three patients with Grade I injury, two with Grade II, and five with Grade IV injury. Among all trauma admissions, the incidence of diagnosed VAI was 0.9% (95% CI 0.5–1.8). Among patients suspected of VAI, there was no univariable association of VAI with C-Spine fracture: OR 4.2 (95% CI 0.51–34.4; *p* = 0.18). There were two (20%) deaths related to VAI. Traumatic VAI was uncommonly detected in this major trauma service in Saudi Arabia. High suspicion and liberal screening by CT angiography in cases where VAI is possible should be considered to avoid missed injuries.

## Introduction

Blunt cerebrovascular injury (BCVI) includes any form of non-penetrating damage to the internal carotid and vertebral arteries^1^. The understanding of BCVI has significantly improved over the past decade of trauma care due to advanced imaging modalities. BCVI includes two clinical entities: vertebral artery injury (VAI) and carotid artery injury. Blunt VAI is an uncommon entity, but important to diagnose with a view to preventing medium to longer-term stroke^2^. VAI presents a clinical challenge since it is difficult to detect, has a diverse presentation, and there are no widely accepted guidelines on diagnosis and management. VAI, although frequently asymptomatic, can have disastrous consequences related to basilar territory infarction and death.

A high index of suspicion for VAI, based on the mechanism of trauma and the nature of associated injuries, should be considered. Cervical spine fractures have been previously reported as being the only independent predictor of VAI^3^. Other potential risk factors include high-energy mechanisms, facial fractures, the base of skull fractures, and diffuse axonal injury with GCS < 6^4^. The reported incidence is highly variable in the literature (0.5–2% of all trauma patients)^5^. For those reasons, there have been several screening criteria set up for the detection of VAI, including the Denver, Memphis, and Boston criteria based on injury mechanism, injury pattern, and symptoms^6–10^. The modified Denver criteria are the most widely used in practice^4^. However, a balance between excess imaging and missed VAI has not been adequately validated in terms of diagnostic accuracy (sensitivity, specificity, positive predictive value, and negative predictive value) and cost-effectiveness. It has been suggested that early diagnosis and management (conservative versus interventional) may improve outcomes.

This study aimed to determine the incidence and nature of VAI in blunt polytrauma patients in a major trauma centre in Saudi Arabia. The secondary aim was to determine variables independently associated with VAI.

## Methods

### Setting

King Saud Medical City (KSMC) is a tertiary care centre in Riyadh with 1400 inpatient beds. In 2018, a total of 36,052 trauma patients presented to the emergency department (ED) of KSMC, of which 3552 patients were admitted. A dedicated trauma unit admits all polytrauma patients.

### Design

This was an observational study based on the retrospective data of all polytrauma patients admitted under the trauma unit between 01 April 2018 and 31 July 2019, who had CT neck angiography to diagnose VAI according to the modified Denver criteria^4^. All blunt polytrauma patients who present to our ED are investigated with whole-body CT as part of the trauma imaging protocol, which includes CT brain, face, cervical spine, chest, abdomen, and pelvis. If the whole-body CT report suggests BCVI according to modified Denver criteria, we investigate further with a neck CT angiogram. We searched the "Carestream Vue Motion" radiology image database used in our institution to identify all patients who underwent CT angiography neck after trauma during the study period. After the selection of these patients, an explicit chart review of medical records was conducted to extract the data.

### Data

Extracted data included demographic details (gender, age), mechanism of injury, injuries of the head, face, & neck, and the CT angiography of neck findings. Furthermore, we extracted data on the modified Denver criteria^4^, such as traumatic brain injury (TBI) with neurologic exam incongruous with head CT scan findings, the base of skull fractures involving the carotid canal, Le Fort fracture type 2 or 3, mandibular fracture, cervical spine fracture, and its pattern, and occipital condyle fracture. If the CT angiography of the neck detected VAI, the grading of injury, the segment of the vertebral artery involved, site of injury, associated vascular injuries, management, and complications, including disability and death, were also extracted.

### Grades of VAI^4^

Radiologically, the VAI is classified into five categories. The Grade I is a mild intimal injury or irregular intima with < 25% luminal narrowing, Grade II is dissection with raised intimal flap/intramural hematoma with luminal narrowing > 25%/intraluminal thrombosis, Grade III is pseudoaneurysm, Grade IV is vessel occlusion/thrombosis, and Grade V is complete transection of the vessel.

### Segments of Vertebral arteries^11^

The vertebral artery is typically divided into four segments: V1 (pre-foraminal) is from the origin to the transverse foramen of C6, V2 (foraminal) is from the transverse foramen of C6 to the transverse foramen of C2, V3 (atlantic, extradural or extraspinal) is from C2 to the dura, and V4 (intradural or intracranial) is from the dura to their confluence to form the basilar artery.

### Treatment and follow up of VAI^1^

**Figure 1:**
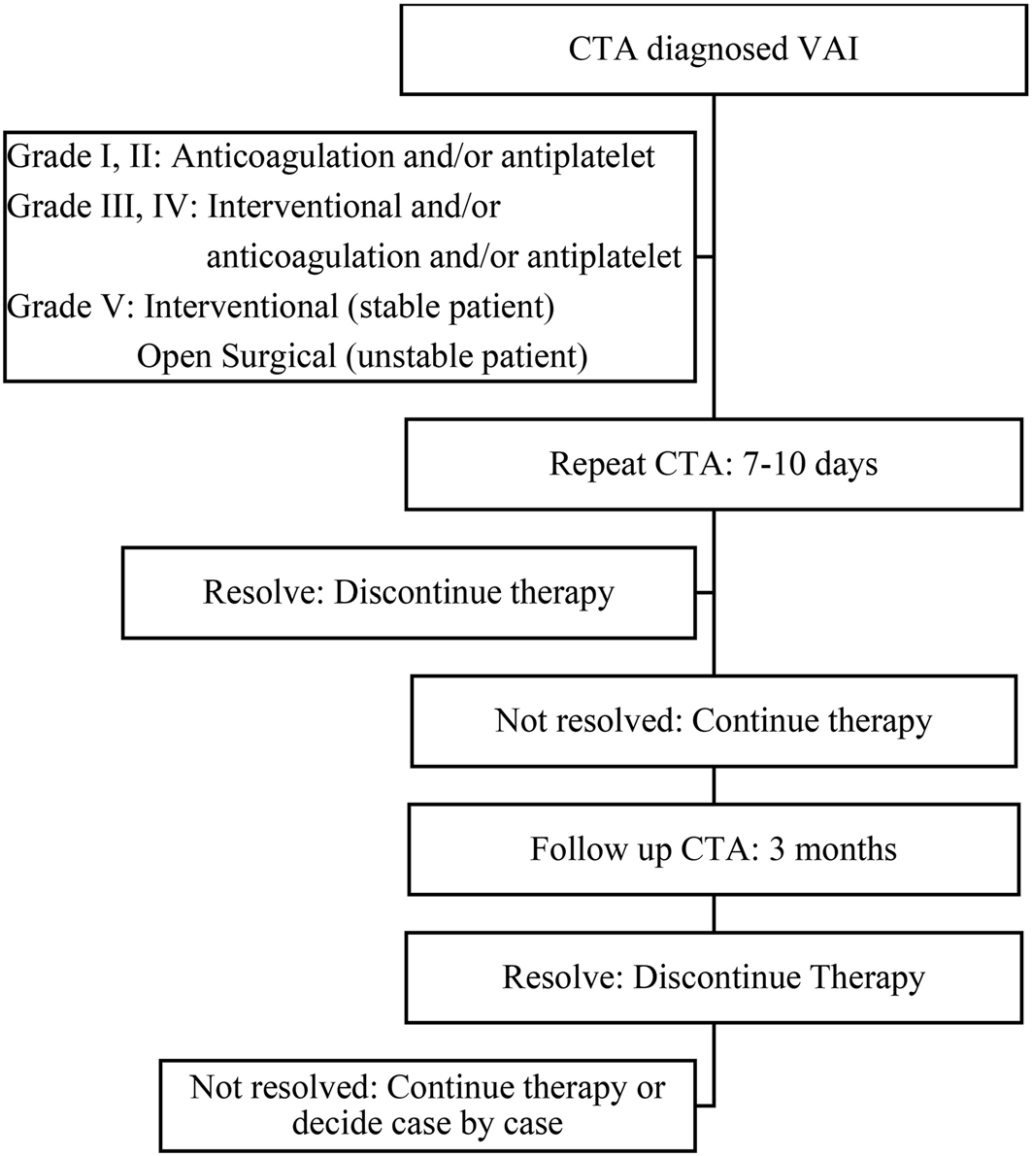
Treatment and follow up of VAI.

The treatment and follow up protocol is described in Fig. 1.

### Analysis

Categorical variables were presented using frequency tables and differences assessed using Fisher's exact test or the chi-square test. Numerical variables were summarised using mean and standard deviation for continuous variables, and for ordinal variables or variables with skewed distribution, median & interquartile range were used. Differences between means were reported using Student's t-test, and the difference between medians reported using the Wilcoxon Rank Sum test, Variables exhibiting some association on univariable analysis (*p* < 0.10) were further assessed using multivariable logistic regression analysis. The performance of the model was assessed using the area under the receiver operator curve. Hosmer–Lemeshow goodness of fit as reported and variance inflation factors were used to assess for multi-collinearity. The independent association of variables with VAI was reported using adjusted OR and 95% confidence intervals. A *p* value of < 0.05 was considered statistically significant. All the analyses were conducted using Stata v 15.1 (College Station, Texas, USA).

The study was approved by the Institutional Review Board (IRB) of the KSMC with a reference number of H1R1-08-Apr19-04.

## Results

**Figure 2:**
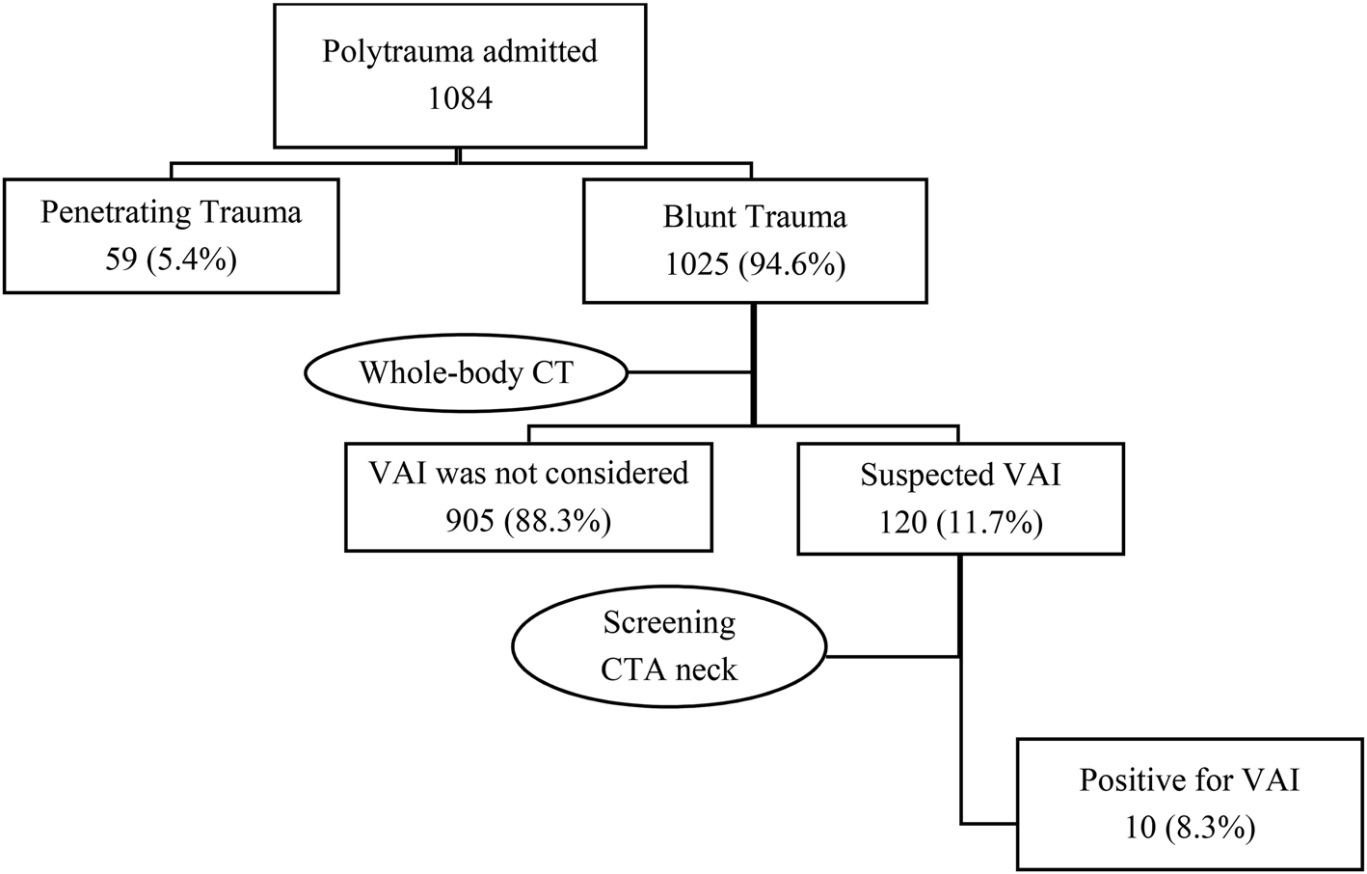
Selection of patients.

A total of 1084 polytrauma patients were admitted during the study period, of which 59 (5.4%) patients were penetrating trauma. Out of 1025 (94.6%) blunt trauma patients, 120 (11.7%) underwent screening CT neck angiography (Fig. 2).

**Figure 3:**
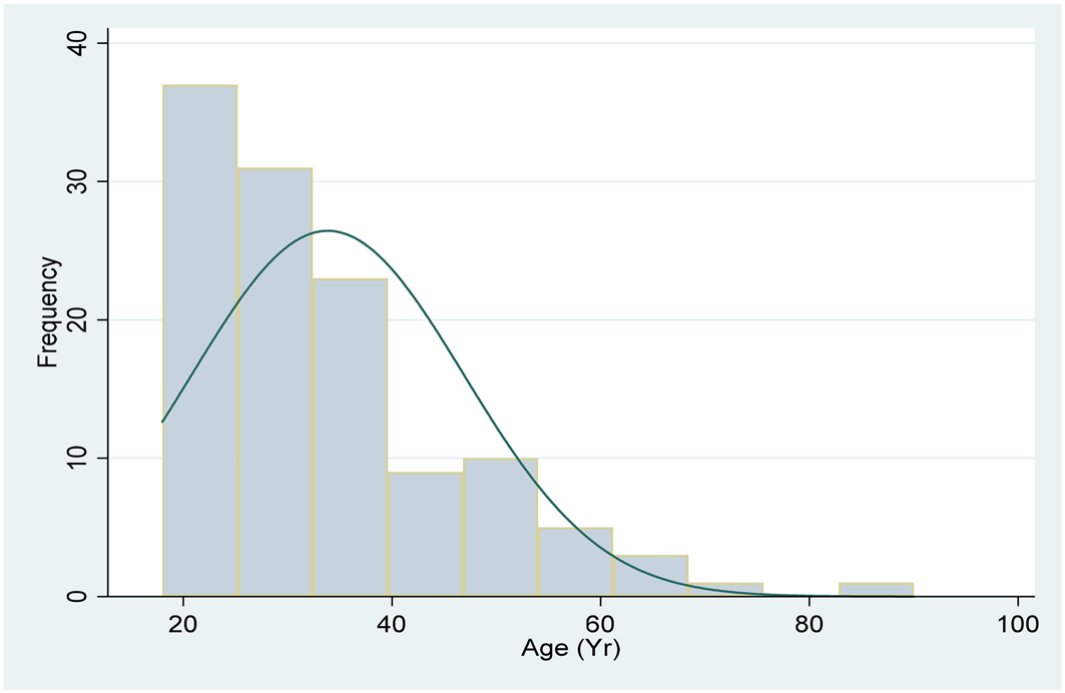
Demographics age distribution.

Demographics were mainly young males with a mean age of 33.8 (SD 13.0) years. The age distribution is presented in Fig. 3.

There were 84 (70%) patients with C-Spine fractures. Among the other indications for CTA neck, according to modified Denver criteria, were traumatic brain injury (TBI) with neurologic exam incongruous with head CT scan findings (n = 46), the base of skull fractures (n = 8), facial fractures (n = 49). Of these, 18, 2, and 19 patients did not have C-Spine fractures, respectively. A comparison of variables, sub-grouped by the diagnosis of C-Spine fractures is presented in Table 1.

**Table 1:** Comparison of patients with suspected VAI with or without C-Spine fractures.

	C-Spine fracture (n = 84)	No C-Spine fracture (n = 36)	*p* value
Age (mean years, SD)	31.7 (12.2)	34.8 (13.3)	0.24
Male sex (%)	74 (88.1%)	31 (86.1%)	0.76
**Mechanism**			0.27
Motor Vehicle Collision (%)	73 (86.9%)	28 (77.8%)	
Falls (%)	10 (11.9%)	7 (19.4%)	
Assault (%)	1 (1.2%)	1 (2.8%)	
Respiratory rate (mean breath/min, SD)	19.7 (2.6)	20.3 (19.4)	0.23
Pulse rate (mean beat/min, SD)	88.2 (19.3)	99.4 (21.9)	0.006
Systolic Blood Pressure (mean mm Hg, SD)	122.8 (20.0)	121.8 (22.6)	0.80
**Glasgow Coma Scale**			0.27
3–8 (%)	1 (1.2%)	1 (2.8%)	
9–12 (%)	10 (11.9%)	7 (19.4%)	
13–15 (%)	73 (86.9%)	28 (77.8%)	
International Normalized Ratio (mean, SD)	1.1 (0.15)	1.1 (0.16)	0.88
**Injury Severity Score**			0.88
0–15 (%)	32 (38.1%)	12 (33.3%)	
16–25 (%)	32 (38.1%)	15 (41.7%)	
> 25 (%)	20 (23.8%)	9 (25%)	
Intubated (%)	39 (46.4%)	27 (75%)	0.004
Blood transfusion (%)	20 (23.8%)	16 (44.4%)	0.024
Traumatic Brain Injury (%)	28 (33.3%)	18 (50%)	0.08
Base of Skull fracture (%)	6 (7.1%)	2 (5.6%)	0.009
Facial fracture (%)	30 (35.7%)	19 (52.8%)	< 0.001

There were 10 (8.3%; 95% CI 4.1–14.8) patients with VAI. Among all trauma presentations, the incidence of VAI was 0.9% (95% CI 0.5–1.8). There was no univariable association of VAI with C-Spine fracture: OR 4.2 (95% CI 0.51–34.4; *p* = 0.18). When adjusted for potential confounders, VAI was not independently associated with any of the potential predictive variables (Table 2), and in particular, when adjusted for other variables, the presence of a C-Spine fracture was not significantly associated with VAI (OR 3.32 (95% CI 0.30–6.2).

**Table 2:** Results of the multivariable logistic regression model.

Variable	Adjusted OR (95% CI)	*p* value
C-Spine fracture	3.32 (0.30–6.2)	0.32
Pulse rate	0.99 (0.96–1.03)	0.91
Intubation	1.05 (0.26–4.15)	0.94
Blood transfusion	2.22 (0.55–9.0)	0.26
Facial fracture	0.48 (0.08–3.0)	0.43

**Figure 4:**
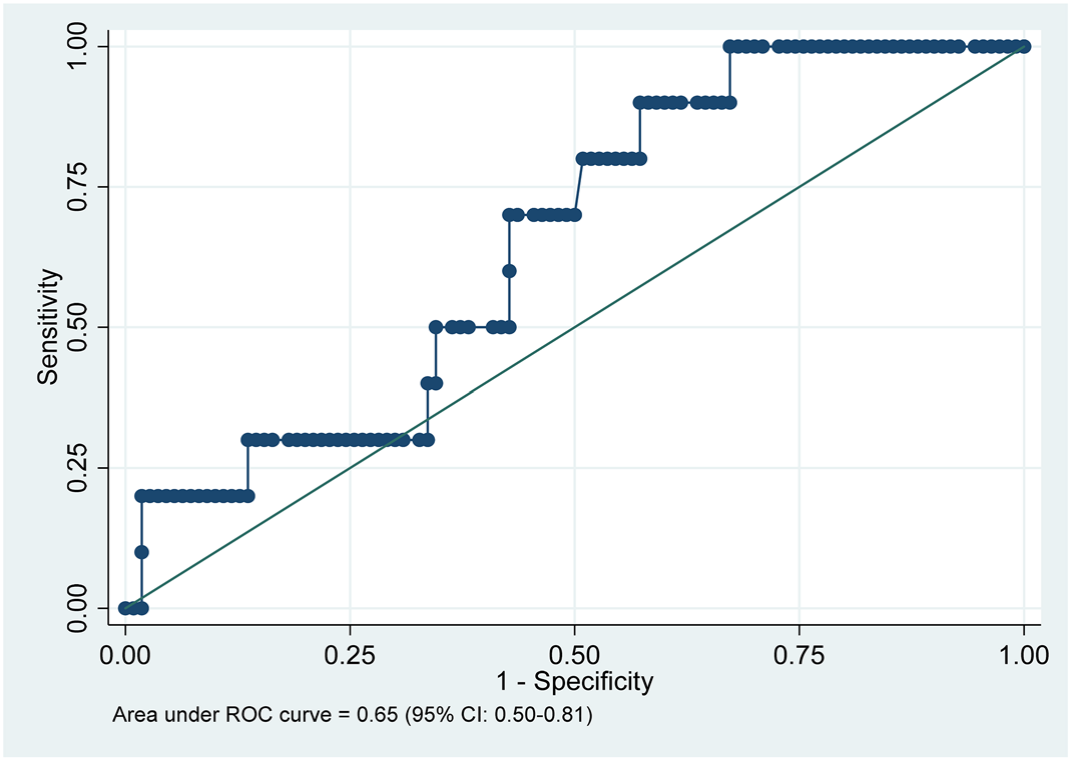
Area under the receiver operating characteristic (AUROC) curve.

The area under the receiver operating characteristic (AUROC) for the model was 0.65 (95% CI 0.50–0.81) (Fig. 4). The *p* value for Hosmer–Lemeshow Goodness of fit was 0.76. Variance inflation factors for all variables were less than 1.6, with a mean VIF of 1.27.

There were three patients with Grade I injury, two with Grade II, and five patients with Grade IV injury. The nature (Table 3), outcome, and follow up at three months (Table 4) of the VAI are described below.

**Table 3:** Nature of the VAI.

Sl. No	Grade of injury	Segment involved	Side of VAI injury	Nature of C-spine fractures	Associated vascular injuries
1	IV	V3	Left	C1 left lateral mass fracture	No
2	IV	V3	Bilateral	C2 right transverse process fracture	Bilateral internal carotid arteries
3	I	V3	Right	C1 right lateral mass fracture	No
4	I	V3	Left	C0 left occipital condyle fracture	No
5	II	V2	Right	C2 right pedicle and transverse process fracture	No
6	IV	V1, V2	Right	C5 right transverse foramen with facet dislocation	No
7	II	V2	Left	C2, C3, C4 left transverse process fractures	No
8	I	V1	Right	C6-C7 fracture-dislocation	No
9	IV	V1, V2, V3	Bilateral	C6, C7 left transverse process fractures	Bilateral Subclavian arteries
10	IV	V1, V2, V3	Right	C6 right foramen transversarium fracture	No

**Table 4:** Outcome, and follow up (at three months) of the VAI.

Sl. No	Grade of injury	Outcome	Follow up CTA neck
1	IV	Discharged	No interval change
2	IV	Death	N/A
3	I	Discharged	Normal
4	I	Discharged	Normal
5	II	Discharged	No interval change
6	IV	Discharged	No interval change
7	II	Discharged	Interval improvement
8	I	Discharged	Normal
9	IV	Death	N/A
10	IV	Discharged	No interval change

There were only two (20%) deaths related to VAI.

The first patient was a 27-year-old female unrestrained front seat passenger involved in high-speed motor vehicle collision sustained severe head, face, neck, and chest trauma. On presentation, she was hemodynamically stable, GCS 3 (intubated, and ventilated), and her ISS was 22. CT angiography neck and brain showed bilateral internal carotid arteries were tapered and blocked entirely in the proximal extracranial portion about 1.8 cm after the origin. Bilateral vertebral arteries were also not showing any distal flow above the C1 level. There was diffuse brain edema with the multiple hypodense areas in the brain with the obliteration of the sulci. She was admitted to ICU and died after five days.

The second patient was a 39-year-old male restrained driver involved in high-speed motor vehicle collision sustained head, neck, and severe chest injuries. On presentation, the patient was hypotensive, GCS 3 (intubated, and ventilated), and his ISS was 29. He responded to fluid resuscitation, and bilateral intercostal drains were inserted for hemo-pneumothoraces. His CT neck and chest angiography demonstrated bilateral subclavian and vertebral arteries injury. There was also right posterior superior mediastinal hematoma with no visible underlying active contrast extravasation. CT brain confirmed bilateral cerebellar and pontomedullary areas of low attenuation, most likely acute ischemic insult. Considering his poor prognosis, the patient was palliated and died after 13 days of ICU stay.

The three Grade I VAI was treated with an antiplatelet agent (low dose Aspirin, 81 mg) alone. The remaining five (Grade II and IV) patients with VAI were treated with an anticoagulant and an antiplatelet agent (low dose Aspirin, 81 mg). Regarding anticoagulant, we initially started Enoxaparin 1 mg/kg subcutaneous injection 12 hourly. On discharge, we converted to oral Apixaban 10 mg bid for one week and then 5 mg bid for the rest of three months duration). All of them had GCS 15 on discharge. In three months follow up at the outpatient department, no further complications or neurological deterioration were observed.

There were no other patients identified with symptoms of VAI at discharge.

## Discussion

The diagnosis of blunt VAI was rare in a high-volume major trauma centre in Saudi Arabia. We were unable to demonstrate independent associations with common risk factors, demonstrating difficulty in the prediction of this condition. In particular, the discriminatory ability for C-Spine fractures screen patients could not be proven. Our results are consistent with previous studies reporting inadequate diagnostic utility of screening tools^12^.

The finding of cervical vertebral fracture involving the foramen transversarium or in its anatomical vicinity in 9 of the 10 cases of VAI suggests a clinically significant finding and a high degree of suspicion to image the vertebral artery in such patients. The only patient who did not have a cervical vertebral fracture had a C0 left occipital condyle fracture. While this is strictly not part of the cervical vertebrae, it is clinically prudent to consider the two occipital condyles and the first cervical vertebra as one functional unit. As such, although statistical significance could not be demonstrated due to the small numbers, there were signals of association of VAI with sub-types of cervical vertebral fractures.

Blunt trauma to the cervical spine can cause injury to the vertebral artery, although no specific cervical vertebral fracture pattern has been associated with VAI^13^. However, the initial presentation of unilateral VAI is usually asymptomatic; only 12–20% of the patients present with ischemic signs and symptoms^14^. Fractures involving the transverse foramen and subluxation are highly associated with VAI by 46–75% of cervical trauma^15^. Bilateral injury to the cerebrovascular arteries occurs in 18–25% of patients with VAI. Only 9 case reports were published regarding blunt trauma to three or four cerebrovascular arteries^16^. The mortality due to blunt carotid injury is 13–38%, whereas the death due to VAI is about 8–18%^17^.

Stroke is the most feared complication of VAI and reported in 10–13% of patients. Therefore, early screening in patients with VAI may decrease the incidence of stroke^18^. Even in cases where antiplatelet agents may be contra-indicated due to concomitant injuries, the advantages of screening for BCVI at the time of presentation aids planning for the treatment, close follow-up, and possible preventing delayed presentation with ischaemic posterior circulation events.

Catheter angiography is the gold standard modality to diagnose VAI, but since it is time-consuming and expensive, thus computerized tomographic angiography (CTA) has become the most common screening method for VAI in acute trauma setting^19^. As described in the literature, the sensitivity of CTA neck to diagnose VAI reaches up to 99%^20^, and it is considered as a modality of choice for diagnosis. Magnetic resonance imaging has shown that satisfactorily results in many studies with the advantage of avoiding contrast. Still, the major disadvantage is a lack of timely availability at many institutions and the incompatibility of ventilatory and orthopaedic fixation equipment with the magnet.

This study is limited in being a retrospective cohort, and only a small sample of patients with VAI were identified. However, it includes consecutive patients during the time period from the most active trauma centre in the country. With only 10 cases of VAI, our attempts to develop a model to predict VAI was grossly underpowered and may suffer from Type II error. There was a signal that c-spine fractures were associated with VAI (odds ratio, OR 3.32), but our confidence in this point estimate was limited due to the small number of cases. While we reported on the hospital outcome of death, functional status, and longer-term functional outcomes of survivors should be the focus of future studies. A national trauma registry with systematic data collection of data on patient outcomes would be invaluable to assess such uncommon but clinically significant injuries. The investigations and association of variables to VAI will require ongoing surveillance using this registry.

## Conclusion

Traumatic VAI was found to be an uncommon entity in the largest major trauma service in Saudi Arabia. Deaths in the setting of diagnosed VAI were uncommon. The association with traditional risk-factors could not be proven. We, therefore, continue to recommend the utilization of local protocols for assessment of VAI and ongoing surveillance for missed injuries.

## Use of experimental animals and human participants

The experiment protocol for involving human data was following the guidelines of national/international/institutional or Declaration of Helsinki in the manuscript. The study was approved by the Institutional Review Board (IRB) of the KSMC with a reference number of H1R1-08-Apr19-04***.*** The IRB committee approved a waiver of the requirement to seek informed consent from the participants for a retrospective review of their data.

## Abbreviations

BCVI: Blunt cerebrovascular injury
VAI: Vertebral artery injury
KSMC: King Saud Medical City
ED: Emergency Department
CT: Computed tomography
CTA: Computed tomography angiography
CI: Confidence interval
OR: Odds ratio
AUROC: Area under the receiver operating characteristic

## References

[1] Brommeland T (2018). Best practice guidelines for blunt cerebrovascular injury (BCVI). Scand. J. Trauma Resusc. Emerg. Med..

[2] Snow H, O’Donohoe T, Martin K, Mitra B (2015). Antithrombotic therapy in blunt cerebrovascular injury—do we need more information?. Trauma.

[3] Wei CW, Montanera W, Selchen D, Lian J, Stevens C, de Tilly LN (2010). Blunt cerebrovascular injuries: diagnosis and management outcomes. Can. J. Neurol. Sci..

[4] Cothren CC, Biffl WL, Moore EE, Kashuk JL, Johnson JL (2009). Treatment for blunt cerebrovascular injuries: equivalence of anticoagulation and antiplatelet agents. Arch. Surg..

[5] Rutman AM, Vranic JE, Mossa-Basha M (2018). Imaging and management of blunt cerebrovascular injury. Radiographics.

[6] Bromberg WJ (2010). Blunt cerebrovascular injury practice management guidelines: the Eastern Association for the Surgery of Trauma. J. Trauma.

[7] Biffl WL (2009). Western Trauma Association critical decisions in trauma: screening for and treatment of blunt cerebrovascular injuries. J. Trauma.

[8] Cothren CC, Moore EE, Ray CE, Johnson JL, Moore JB, Burch JM (2007). Cervical spine fracture patterns mandating screening to rule out blunt cerebrovascular injury. Surgery.

[9] Miller PR (2002). Prospective screening for blunt cerebrovascular injuries: analysis of diagnostic modalities and outcomes. Ann. Surg..

[10] Buch K (2016). Association between cervical spine and skull-base fractures and blunt cerebrovascular injury. Eur. Radiol..

[11] Desouza RM, Crocker MJ, Haliasos N, Rennie A, Saxena A (2011). Blunt traumatic vertebral artery injury: a clinical review. Eur. Spine J..

[12] Winn A, Durso AM, Lopera CR, Munera F (2018). Blunt craniocervical trauma: does the patient have a cerebral vascular injury?. Neuroimaging Clin. N. Am..

[13] Biffl WL (2000). The Devastating potential of blunt vertebral arterial injuries. Ann. Surg..

[14] Jang JW, Lee JK, Hur H, Seo BR, Lee JH, Kim SH (2011). Vertebral artery injury after cervical spine trauma: A prospective study using computed tomographic angiography. Surg. Neurol. Int..

[15] Nakao Y, Terai H (2014). Embolic brain infarction related to posttraumatic occlusion of vertebral artery resulting from cervical spine injury: a case report. J. Med. Case Rep..

[16] Ariyada K (2019). Bilateral internal carotid and left vertebral artery dissection after blunt trauma: a case report and literature review. Neurol. Med. Chir. (Tokyo).

[17] Bensch FV, Varjonen EA, Pyhältö TT, Koskinen SK (2019). Augmenting Denver criteria yields increased BCVI detection, with screening showing markedly increased risk for subsequent ischemic stroke. Emerg. Radiol..

[18] Nagpal P, Policeni BA, Bathla G, Khandelwal A, Derdeyn C, Skeete D (2018). Blunt cerebrovascular injuries: advances in screening, imaging, and management trends. Am. J. Neuroradiol..

[19] Timpone V, Schneider BE, Sherman PM (2013). Screening CT angiography for detection of blunt carotid and vertebral artery injury in the setting of combat-related trauma. Mil. Med..

[20] Eastman AL, Chason DP, Perez CL, McAnulty AL, Minei JP (2006). Computed tomographic angiography for the diagnosis of blunt cervical vascular injury: is it ready for primetime?. J. Trauma.

